# Supplementary material for: Comparative multiomics analysis of cell physiological state after culture in a basket bioreactor
Source: Sci Rep. 2022 Nov 23;12:20161. doi: 10.1038/s41598-022-24687-4 (PMC9686226; doi:10.1038/s41598-022-24687-4)
Supplement: Supplementary file 1 — Supplementary Information 1. [file 41598_2022_24687_MOESM1_ESM.zip › raw data/Transcriptomic raw data/6.Enrichment/1.GO/TE1vsTC1/UP/TE1vsTC1.up_GObar.pdf]

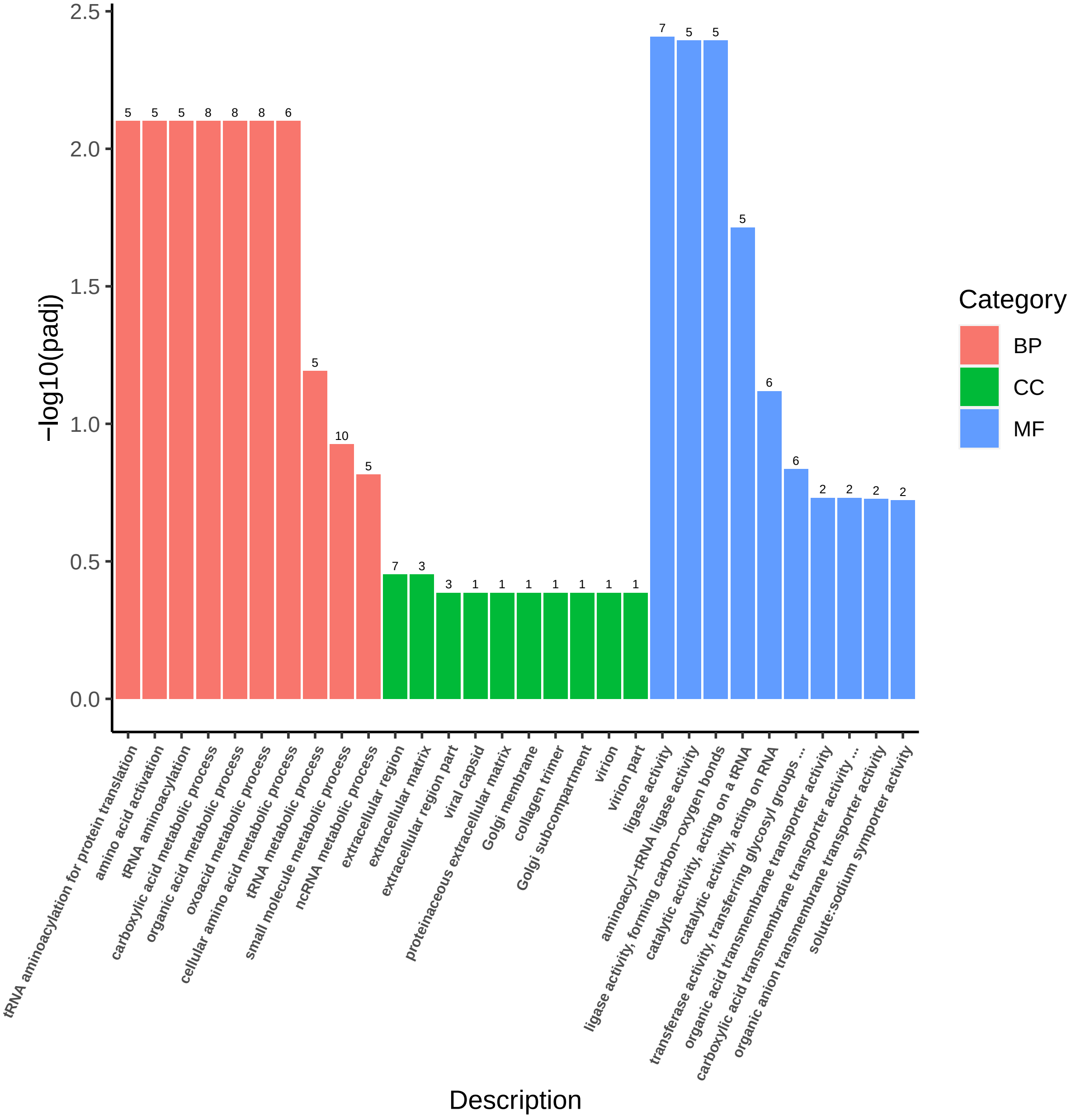

−log10(padj)
2.5
2.0
1.5
1.0
0.5
0.0
5
5
5
8
8
8
6
5
10
5
7
3
3
1
1
1
1
1
1
1
7
5
5
5
6
6
2
2
2
2
tRNA aminoacylation for protein translation
amino acid activation
tRNA aminoacylation
carboxylic acid metabolic process
organic acid metabolic process
oxoacid metabolic process
cellular amino acid metabolic process
tRNA metabolic process
small molecule metabolic process
ncRNA metabolic process
extracellular region
extracellular matrix
extracellular region part
viral capsid
proteinaceous extracellular matrix
Golgi membrane
collagen trimer
Golgi subcompartment
virion
virion part
ligase activity
aminoacyl−tRNA ligase activity
ligase activity, forming carbon−oxygen bonds
catalytic activity, acting on a tRNA
catalytic activity, acting on RNA
transferase activity, transferring glycosyl groups ...
organic acid transmembrane transporter activity
carboxylic acid transmembrane transporter activity ...
organic anion transmembrane transporter activity
solute:sodium symporter activity
Description
Category
BP
CC
MF
